# Missed Opportunities for Providing Low-Fat Dietary Advice to People With Diabetes

**DOI:** 10.5888/pcd9.120086

**Published:** 2012-11-01

**Authors:** Ingrid E. Lobo, Danielle F. Loeb, Vahram Ghushchyan, Irene E. Schauer, Amy G. Huebschmann

**Affiliations:** Author Affiliations: Danielle F. Loeb, University of Colorado School of Medicine, Aurora, Colorado; Vahram Ghushchyan, University of Colorado School of Pharmacy, Aurora, Colorado; Irene E. Schauer, Amy G. Huebschmann, University of Colorado School of Medicine and University of Colorado Center for Women’s Health Research, Aurora, Colorado.

## Abstract

**Introduction:**

Because cardiovascular disease is closely linked to diabetes, national guidelines recommend low-fat dietary advice for patients who have cardiovascular disease or are at risk for diabetes. The prevalence of receiving such advice is not known. We assessed the lifetime prevalence rates of receiving low-fat dietary advice from a health professional and the relationship between having diabetes or risk factors for diabetes and receiving low-fat dietary advice.

**Methods:**

From 2002 through 2009, 188,006 adults answered the following question in the Medical Expenditure Panel Survey: “Has a doctor or other health professional ever advised you to eat fewer high-fat or high-cholesterol foods?” We assessed the association between receiving advice and the following predictors: a diabetes diagnosis, 7 single risk factors for type 2 diabetes, and total number of risk factors.

**Results:**

Among respondents without diabetes or risk factors for diabetes, 7.4% received low-fat dietary advice; 70.6% of respondents with diabetes received advice. Respondents with diabetes were almost twice as likely to receive advice as respondents without diabetes or its risk factors. As the number of risk factors increased, the likelihood of receiving low-fat dietary advice increased. Although unadjusted advice rates increased during the study period, the likelihood of receiving advice decreased.

**Conclusion:**

Although most participants with diabetes received low-fat dietary advice, almost one-third did not. Low-fat dietary advice was more closely associated with the total number of diabetes risk factors than the presence of diabetes. Increasing rates of diabetes and diabetes risk factors are outpacing increases in provision of low-fat dietary advice.

## Introduction

Cardiovascular disease (CVD) is the leading cause of death in the United States (1). The risk of CVD has been associated with the intake of total fat, saturated fat, and trans fat (2,3). The American Heart Association (AHA) and the American Diabetes Association (ADA) recommend people with diabetes consume a low-fat diet (4). The ADA also recommends reduced intake of dietary fat in people at risk for developing type 2 diabetes (5).

The AHA and ADA recommend dietary counseling because even brief advice by physicians can improve dietary behavior (6–9). Despite recommendations, dietary counseling is included in less than half of nonacute primary care visits for patients with diabetes and diabetes risk factors (5,6,10–13).

The role of multimorbidity — multiple concurrent chronic conditions — in guideline-adherent preventive care of diabetes is unclear. Some research suggests worse guideline-adherent preventive care in patients who have multiple chronic conditions because of competing demands (14–16). Other studies demonstrate the opposite, especially when the multimorbid conditions require similar management (17–19). A prominent disease cluster is the cardiometabolic disease cluster of diabetes and risk factors for type 2 diabetes; this disease cluster is likely to be associated with higher rates of reported low-fat dietary advice.

The primary objective of this study was to examine rates of receiving low-fat dietary advice from a health professional. We hypothesized that rates are greater among people who have diabetes or risk factors for diabetes than among people with neither diabetes nor its risk factors. The secondary objective was to determine the relationship between having diabetes or risk factors for diabetes and receiving low-fat dietary advice.

## Methods

### Data source

The Medical Expenditure Panel Survey (MEPS) is a nationally representative survey of the US civilian, noninstitutionalized population conducted since 1996 by the Agency for Healthcare Research Quality and the National Center for Health Statistics. Details of the MEPS data collection process are available (20). Briefly, participants in the MEPS are selected from the National Health Interview Survey. We used the Household Component of MEPS (MEPS-HC). MEPS-HC collects data on demographics, health insurance, and other health-related items from household members, who are surveyed during 2 full calendar years. The sample design of MEPS-HC includes stratification, clustering, multiple stages of selection, and disproportionate sampling (21). MEPS collects supplemental information on responses from the MEPS-HC through a medical provider component, consisting of objective information from hospitals, pharmacies, and medical providers. MEPS maps medical *International* *Classification of Diseases, 9th Revision, Clinical Modification* [ICD-9-CM] codes on the basis of medical and pharmacy use and self-report. To represent the noninstitutionalized US population, MEPS uses sample weights to adjust for factors related to survey design and underresponse (21).

### Study population and ascertainment of characteristics

The study sample was 188,006 MEPS respondents from 2002 through 2009. The mean response rate during this period was 60.9% (range, 56.9%–64.7%) (22). MEPS was reviewed and approved by the Westat institutional review board, established under a multiproject assurance (M-1531) granted by the Office for Protection from Research Risks.

All respondents aged 18 years or older were asked if they had ever received low-fat dietary advice from a health professional: “Has a doctor or other health professional ever advised you to eat fewer high-fat or high-cholesterol foods?” We used the term “low-fat dietary advice” to refer to participants’ response to this question. We used MEPS data to determine whether a respondent had diabetes or risk factors for developing type 2 diabetes. Respondents who answered yes to the following question on the MEPS-HC were classified as having diabetes: “Have you ever been told by a doctor or health professional that you have diabetes?” Similar to other epidemiologic surveys, MEPS does not differentiate between type 1 and type 2 diabetes. Because the Centers for Disease Control and Prevention estimates that more than 90% of adults who have diabetes have type 2 diabetes (23), we assumed that most of our sample who had diabetes had type 2 diabetes.

We used the most recent ADA criteria (10) to classify risk factors for type 2 diabetes. The 7 ADA-designated risk factors represented in the MEPS survey were an age of 45 years or older, Hispanic ethnicity or nonwhite race, body mass index (BMI) of 25.0 kg/m^2^ or more, physical inactivity, hypertension (ICD-9-CM 401), hyperlipidemia (ICD-9-CM 272), and a history of CVD. Age, race, and ethnicity were self-reported. We calculated BMI from self-reported height and weight. Physical inactivity was determined by a negative response to the question, “Do you spend half an hour or more in moderate or vigorous physical activity at least 3 times a week?” Hypertension and hyperlipidemia were designated by the ICD-9-CM code on the MEPS medical provider component. A history of CVD was designated by participants’ positive response to the question on whether they had ever been diagnosed with any of the following: coronary heart disease, angina, heart attack, or stroke.

### Statistical analysis

We performed a cross-sectional analysis of data from the 2002–2009 MEPS. To adjust for the complex sample design and ensure nationally representative estimates, we used MEPS person-level and variance-adjustment weights using Stata version 11 (StataCorp LP, College Station, Texas) for all analyses. We conducted χ^2^ tests to compare rates of receiving low-fat dietary advice among selected subgroups. Multivariate logistic regression analysis was used to estimate the adjusted odds of receiving low-fat dietary advice by the following factors: demographic characteristics (sex, age, race, ethnicity, geographic region, education, and income), general health-related characteristics (health insurance and smoking status), diabetes status, single risk factors for type 2 diabetes, and cardiometabolic multimorbidity (ie, total number of risk factors for type 2 diabetes). For income, we grouped survey respondent by the federal poverty index developed by MEPS (24). In separate regression analyses, independent variables included demographic and general health-related characteristics, diabetes status, risk factors for type 2 diabetes, dummy independent variables representing the number of ADA-designated risk factors for type 2 diabetes (range, 0–7), a modified clinical comorbidity index, and dummy variables for each study year, using 2002 as the reference year (25). The dummy independent variables for the risk factors for type 2 diabetes indicated the total number of risk factors for each respondent (ie, 1 risk factor, 2 risk factors, and so on, up to a maximum of 7 risk factors). The modified clinical comorbidity index represented the number of chronic conditions other than the comorbidities of diabetes or risk factors for diabetes included in the regression model (25). We also examined trends in the unadjusted rates and adjusted likelihood of receiving advice during the study period. We calculated adjusted odds ratios and 95% confidence intervals (CIs). Significance was set at *P* < .05.

## Results

The following demographic factors were related to a greater likelihood of receiving low-fat dietary advice (Table 1): age of 45 or older, a high school degree or more, middle or high income, Hispanic ethnicity, black race, having health insurance, not currently smoking, and residing in the Northeast. Each age group aged 45 or older was more likely to receive low-fat dietary advice than the group aged younger than 45. Sex was not related to the likelihood of receiving low-fat dietary advice. The unadjusted rates of advice increased from 30.6% in 2002 to 35.6% in 2009. The likelihood of receiving low-fat dietary advice decreased from 2004 to 2009, compared with the reference year 2002 (adjusted odds ratio [AOR] in 2004, 0.97; AOR in 2009, 0.88).

**Table 1:** Rates and Likelihood of Receiving Low-Fat Dietary Advice From a Health Professional, by Selected Characteristics, Medical Expenditure Panel Survey, 2002–2009

Characteristic	Unweighted No. of Respondents (N = 188,006)^a^	Unadjusted Advice Rate, % (SE)	Adjusted OR (95% CI)^b^
**Sex**			
Male	86,246	33.0 (0.31)	1 [Reference]
Female	101,760	33.1 (0.29)	1.01 (0.98–1.04)
**Age group, y**			
<45	98,644	19.8 (0.24)	1 [Reference]
45–54	35,092	40.3 (0.45)	1.75 (1.68–1.83)
55–64	25,368	50.5 (0.51)	1.91 (1.82–2.02)
≥65	28,902	49.6 (0.54)	1.35 (1.24–1.44)
**Ethnicity**			
Non-Hispanic	142,785	33.7 (0.27)	1 [Reference]
Hispanic	45,221	28.9 (0.54)	1.32 (1.23–1.40)
**Race**			
White	143,142	33.4 (0.28)	1 [Reference]
Black	30,603	33.0 (0.47)	1.09 (1.03–1.15)
American Indian	1,699	33.7 (1.85)	0.99 (0.82–1.20)
Other	12,562	27.9 (0.76)	1.09 (1.00–1.19)
**Geographic region**			
Northeast	28,308	34.9 (0.54)	1 [Reference]
South	72,673	33.7 (0.41)	0.92 (0.86–0.99)
Midwest	37,504	32.8 (0.60)	0.87 (0.81–0.94)
West	49,521	30.8 (0.45)	0.88 (0.82–0.95)
**Education**			
<High school diploma	46,703	30.1 (0.44)	1 [Reference]
High school diploma	90,248	32.8 (0.31)	1.10 (1.05–1.15)
Some college (<4 y)	12,645	35.8 (0.65)	1.26 (1.17–1.36)
College degree (4 y)	25,008	33.3 (0.46)	1.29 (1.22–1.38)
Some graduate school	12,140	37.5 (0.66)	1.36 (1.25–1.47)
**Income**			
Poor (<100% FPI)	31,354	28.8 (0.44)	1 [Reference]
Near poor (100% to <125% FPI)	10,916	31.6 (0.71)	0.95 (0.87–1.04)
Low income (125% to <200% FPI)	30,494	30.5 (0.47)	1.00 (0.94–1.06)
Middle income (200% to <400% FPI)	56,263	31.7 (0.35)	1.07 (1.02–1.13)
High income (≥400% FPI)	58,979	36.2 (0.35)	1.24 (1.16–1.32)
**Health insurance**			
Private	115,225	34.4 (0.28)	1 [Reference]
Public	36,224	40.5 (0.47)	0.97 (0.92–1.02)
Uninsured	36,557	19.0 (0.43)	0.68 (0.64–0.73)
**Smoking status**			
Not current smoker	130,543	34.9 (0.29)	1 [Reference]
Current smoker	33,741	28.1 (0.38)	0.90 (0.86–0.94)
**Study year**			
2002	26,838	30.6 (0.42)	1 [Reference]
2003	23,014	31.9 (0.45)	1.02 (0.98–1.07)
2004	23,295	32.1 (0.50)	0.97 (0.91–1.04)
2005	23,012	32.4 (0.50)	0.96 (0.90–1.02)
2006	23,252	32.7 (0.50)	0.96 (0.90–1.02)
2007	21,173	34.4 (0.54)	0.93 (0.87–1.00)
2008	22,378	34.6 (0.52)	0.80 (0.75–0.86)
2009	25,044	35.6 (0.57)	0.88 (0.81–0.95)

Abbreviations: SE, standard error; OR, odds ratio; CI, confidence interval; FPI, federal poverty index as defined by Medical Expenditure Panel Survey (24).

^a^ Not all categories add to total number of respondents because of nonresponse to survey item; nonresponse rates were less than 1% for all categories, except smoking status, which had a nonresponse rate of 13%.

^b^ Adjustment covariates were age, sex, race, ethnicity, education, federal poverty index, geographic region, health insurance, smoking status, diabetes, risk factors for type 2 diabetes, and the modified clinical comorbidity index (25).

The likelihood of receiving low-fat dietary advice was also related to diabetes status and other risk factors for type 2 diabetes (Table 2). Among respondents who had diabetes, the unadjusted rate of receiving advice was 70.6%; the rate among respondents who did not have diabetes or risk factors for diabetes was 7.4%, and the rate among respondents who did not have diabetes but had at least 1 risk factor was 32.4%. Respondents who had diabetes were almost twice as likely to receive low-fat dietary advice as respondents who did not have diabetes (controlling for type and number of risk factors for diabetes). Unadjusted rates of low-fat dietary advice ranged according to type of risk factor — from 34.3% (BMI 25.0–29.9) to 75.9% (hyperlipidemia).

**Table 2:** Rates and Likelihood of Receiving Low-Fat Dietary Advice From a Health Professional, by Diabetes Status and Risk Factors for Type 2 Diabetes, Medical Expenditure Panel Survey, 2002–2009

Characteristic	Unweighted No. of Respondents (N = 188,006)^a^	Unadjusted Advice Rate, % (SE)	Adjusted OR (95% CI)^b^
**Diabetes status**			
No diabetes or risk factors	13,383	7.4 (0.31)	1 [Reference]
No diabetes but ≥1 risk factor	153,580	32.4 (0.27)	
Diabetes	15,857	70.6 (0.58)	1.91 (1.78–2.05)
**Risk factors for type 2 diabetes^c^**			
**Body mass index (kg/m^2^)**			
Underweight (<18.5)	3,507	12.5 (0.76)	0.64 (0.55–0.74)
Normal weight (18.5–24.9)	63,023	19.6 (0.25)	1 [Reference]
Overweight (25.0–29.9)	64,468	34.3 (0.34)	1.83 (1.77–1.90)
Obese (≥30.0)	52,553	50.6 (0.40)	3.46 (3.32–3.62)
**Physically active**			
No	101,620	30.2 (0.28)	1 [Reference]
Yes	85,726	37.0 (0.34)	1.01 (0.98–1.05)
**Hypertension**			
No	146,998	25.3 (0.24)	1 [Reference]
Yes	41,008	60.5 (0.42)	1.59 (1.53–1.67)
**Hyperlipidemia**			
No	161,262	25.1 (0.23)	1 [Reference]
Yes	26,744	75.9 (0.45)	4.85 (4.60–5.12)
**Cardiovascular disease**			
No	173,089	30.3 (0.24)	1 [Reference]
Yes	14,678	65.0 (0.61)	1.59 (1.48–1.70)

Abbreviations: SE, standard error; OR, odds ratio; CI, confidence interval.

^a^ Not all categories add to total number of respondents because of nonresponse to survey item; nonresponse rates were less than 3% for all categories.

^b ^Adjustment covariates were age, sex, race, ethnicity, education, federal poverty index, geographic region, health insurance, smoking status, diabetes, risk factors for type 2 diabetes, and the modified clinical comorbidity index (25).

^c^ The risk factors designated by the American Diabetes Association and represented in the Medical Expenditure Panel Survey were age of 45 years or older, Hispanic ethnicity or nonwhite race, body mass index of 25.0 or more, physical inactivity, hypertension (*International Classification of Diseases, 9th Revision, Clinical Modification* [ICD-9-CM] 401), hyperlipidemia (ICD-9-CM 272), and a history of cardiovascular disease.

Some risk factors for type 2 diabetes were more closely related to low-fat dietary advice than others. Respondents who had hyperlipidemia were almost 5 times as likely to receive advice as respondents who did not have this risk factor. Obese respondents were 3.5 times as likely to receive advice as normal-weight respondents. Respondents who had CVD were 1.6 times as likely to receive advice as respondents who did not have CVD.

Participants with 1 risk factor for type 2 diabetes were more than twice as likely to receive low-fat dietary advice as respondents with no diabetes and no risk factors (Figure). As the number of risk factors increased, the likelihood of receiving low-fat dietary advice increased.

**Figure:**
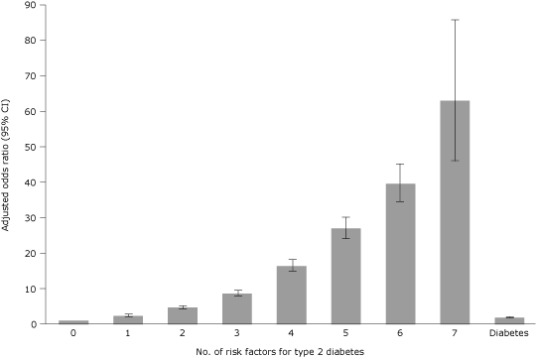
Likelihood of receiving low-fat dietary advice, by number of risk factors for type 2 diabetes and by diabetes status, Medical Expenditure Panel Survey, 2002–2009 (n = 188,006). Adjustment covariates were population characteristics (age, sex, race, ethnicity, education, federal poverty index, and geographic region), smoking status, dummy variables for each potential number of diabetes risk factors, and the modified clinical comorbidity index (25). The reference group includes respondents who had no diabetes and no risk factors for diabetes type 2. Error bars represent 95% confidence intervals. **Table:** 

No. of Risk Factors	Odds Ratio (95% Confidence Interval)
0	1.0 [Reference]
1	2.4 (2.2–2.7)
2	4.7 (4.2–5.1)
3	8.6 (7.8–9.5)
4	16.4 (14.8–18.2)
4	27.0 (24.1–30.2)
6	39.6 (34.6–45.2)
7	63.0 (46.2–85.9)
Diabetes	1.9 (1.8–2.1)

## Discussion

In this nationally representative study of participants in the MEPS survey, we found that respondents who had diabetes or ADA-designated risk factors for type 2 diabetes were more likely to receive low-fat dietary advice than respondents who had neither diabetes nor diabetes risk factors. However, almost one-third of respondents who had diabetes and 24% to 68% of respondents who had risk factors for type 2 diabetes did not receive advice. Risk factors for type 2 diabetes that are also major cardiovascular risk factors were strongly associated with low-fat dietary advice, including hyperlipidemia and obesity. As cardiometabolic multimorbidity increased, the likelihood of receiving low-fat dietary advice also increased.

Brief dietary counseling in primary care settings improves dietary behavior, weight, and lipid levels (6–8). Primary care providers deliver nutrition counseling in less than half of their patient visits, even for patients with diabetes or chronic disease (5,6,10–13). Our study showed that 70.6% of respondents with diabetes ever received low-fat dietary advice from a health professional. To our knowledge, our study is the first nationally representative study to identify the lifetime prevalence of receiving low-fat dietary advice from a health professional in people with diabetes or risk factors for diabetes.

Our data demonstrate missed opportunities to provide low-fat dietary advice for almost one-third of people with diabetes. Additionally, respondents who had diabetes were only twice as likely to receive advice as respondents who had no diabetes or risk factors or respondents with 1 risk factor. Respondents who had greater cardiometabolic multimorbidity (up to 7 risk factors) were far more likely to receive advice.

The discrepancy between the high unadjusted rate (70.6%) and only modestly increased likelihood of low-fat dietary advice in respondents who had diabetes compared with respondents who did not have diabetes is likely related to our adjustment for cardiometabolic multimorbid conditions, such as obesity and hyperlipidemia, in patients who had type 2 diabetes. Receiving low-fat dietary advice seems more strongly related to cardiometabolic multimorbidity than the presence of diabetes. These findings suggest that people with diabetes and a normal BMI and normal lipid profiles — a common profile of patients with type 1 diabetes — would be less likely to receive low-fat dietary advice than people with diabetes and other comorbidities. This possibility is a concern because people with type 1 diabetes have a high risk of CVD and consume higher-than-recommended levels of saturated fats (26).

The unadjusted rates of low-fat dietary advice increased during the study period while the adjusted likelihood of advice decreased. These discrepant trends indicate that increasing rates of diabetes and diabetes risk factors during the last decade (23) are outpacing the increasing receipt of low-fat dietary advice. In our study, the rates of low-fat dietary advice were greatest in respondents with diabetes or risk factors for type 2 diabetes. Possible reasons for not advising patients include lack of confidence that provider-delivered dietary advice will be effective, insufficient time and reimbursement, and a lack of infrastructure to support dietary education by staff (6,11). Competing demands such as the myriad evidence-based diabetes management goals may challenge providers’ abilities to offer low-fat dietary advice (27).

In contrast to this “competing demands” argument, we found that the likelihood of low-fat dietary advice increased with the number of diabetes risk factors, indicating an association between cardiometabolic multimorbidity and guideline-adherent dietary advice from providers. Greater multimorbidity has been associated with greater quality-of-care scores; physicians may be more likely to meet evidence-based recommendations for patients who have greater multimorbidity and a greater perceived need for improved care (17,18). Our findings also reinforce the argument that providers are more likely to offer therapies that are indicated for patients who have multiple conditions that warrant similar treatments, termed “concordant conditions.” In contrast, chronic conditions that warrant different treatments are termed “discordant conditions” and appear to create conflicting priorities for clinicians that may impede evidence-based treatment (19).

Consistent with the strength of the relationship between a low-fat diet and levels of total and low-density-lipoprotein cholesterol, we found that hyperlipidemia was the strongest single clinical predictor of receiving low-fat dietary advice (28). The weaker association between low-fat dietary advice and other diabetes risk factors may be partly related to the current complex dietary recommendations for people with diabetes and at risk for diabetes. In addition to limiting fat intake, the ADA also recommends limiting carbohydrate intake, eating high-fiber foods, and limiting consumption of alcohol, sweeteners, and protein (5). When charged with a litany of dietary messages to give to patients with diabetes, health providers may focus their dietary advice more on carbohydrate restriction and less on fat restriction.

Although evidence-based guidelines clearly advise a low-fat diet to reduce CVD risk for patients with diabetes and risk factors for diabetes, these patients would benefit from comprehensive, individualized nutritional advice from a dietitian. Providers may also benefit from clearer guidance and succinct educational materials such as the ADA-endorsed “plate method” diagram (29). In 2011, the US Department of Agriculture (USDA) also endorsed a similar plate method that emphasizes portion control while also recommending lean proteins and fat-free or low-fat dairy products (30). When possible, providers should also refer patients for ADA-certified diabetes education to allow more in-depth dietary education (5).

The principal strength of this study is the external validity provided by the nationally representative population-based sampling techniques used in the MEPS database. Limitations include self-report of low-fat dietary advice. Self-reported conditions are likely to be underreported (31); thus, our results may underestimate the likelihood of low-fat dietary advice. Also, the type of low-fat dietary advice recalled by respondents may have varied in intensity — from simple, directive advice to eat a low-fat diet to formal dietary counseling. Dietary intake was not assessed in this study, and future research is needed to determine whether clinician dietary advice affects diet. This analysis did not differentiate between type 1 and type 2 diabetes. Because more than 90% of people who have diabetes have type 2 diabetes (23), our results most accurately describe people with type 2 diabetes.

Recognizing the strong link between CVD and diabetes and the correlation of fat intake with CVD risk, the AHA and ADA recommend low-fat dietary counseling. Health professionals are missing opportunities to provide low-fat dietary advice to people with diabetes and at risk for diabetes. Our research indicates that professionals are not meeting the public health nutritional counseling goal of advising all patients with diabetes or at risk for diabetes to eat a low-fat diet. This research emphasizes the importance of developing and testing optimal methods of delivering medical nutrition counseling. Future studies should also focus on the effects of the USDA- and ADA-endorsed plate methods, which are simple to explain. They reinforce nutritional recommendations to follow a diet that restricts consumption of fats and carbohydrates, promotes consumptions of vegetables, and encourages portion control.
